# POSTPANDEMIC SUCCESSES AND CHALLENGES IN REOPENING OLDER AMERICANS ACT CONGREGATE MEALS

**DOI:** 10.1093/geroni/igad104.0292

**Published:** 2023-12-21

## Abstract

The COVID-19 pandemic required Older Americans Act (OAA) congregate nutrition programs to shut down in-person dining but continue serving meals in innovative ways. Re-opening provided a unique opportunity for congregate nutrition programs to continue these innovative changes and/or reinvent how they serve meals. These changes will be crucial for OAA nutrition programs to maintain their relevance, as all adults over age 60 qualify for OAA meals, yet fewer than that attend. To identify successful practices that could be adopted nationally and describe continuing challenges, we conducted surveys, focus groups, and interviews of congregate nutrition programs. Overall, 523 completed the entire survey, nine participated in focus groups, and three were interviewed. Responses came from across 47 states with most (94%) reporting permanently adopting service delivery methods implemented during the COVID-19 pandemic. Overall, nutrition programs described grab-and-go meals having attracted new participants during the pandemic and programs pairing other services with meals to appeal to and retain a wide range of participants. These include medically tailored meals, culturally relevant meals, partnerships with other organizations, and entertainment. These results can be used to strengthen congregate nutrition programs.

